# CRISPR-mediated gene targeting of CK1δ/ε leads to enhanced understanding of their role in endocytosis via phosphoregulation of GAPVD1

**DOI:** 10.1038/s41598-020-63669-2

**Published:** 2020-04-22

**Authors:** Rodrigo X. Guillen, Janel R. Beckley, Jun-Song Chen, Kathleen L. Gould

**Affiliations:** 1 Department of Cell and Developmental Biology, Vanderbilt University School of Medicine, Nashville, TN 37232 USA; 2 Present Address: Calico Group LLC, ProteoWorker, Nashville, TN 32712 USA

**Keywords:** Biochemistry, Cell biology, Post-translational modifications, Proteomics

## Abstract

Human casein kinase 1 delta (CK1δ) and epsilon (CK1ε) are members of a conserved family of abundant, ubiquitously expressed serine/threonine kinases that regulate multiple cellular processes including circadian rhythm and endocytosis. Here, we have investigated the localization and interactomes of endogenously tagged CK1δ and CK1ε during interphase and mitosis. CK1δ and CK1ε localize to centrosomes throughout the cell cycle, and in interphase cells to the nucleus, and in both a diffuse and punctate pattern in the cytoplasm. Also, for the first time, they were detected at the midbody during cell division. Mass spectrometry analysis identified a total of 181 proteins co-purifying with a Venus multifunctional (VM)-tagged CK1δ and/or CK1ε. GTPase-activating protein and VPS9 domain-containing protein 1 (GAPVD1), a protein required for efficient endocytosis, was consistently one of the most abundant interacting partners. We demonstrate that GAPVD1 is a substrate of CK1δ/ε with up to 38 phosphorylated residues *in vitro* and *in vivo*. Wildtype and a phosphomimetic mutant of GAPVD1, but not a phospho-ablating mutant, were able to rescue defects in transferrin and EGF internalization caused by loss of endogenous GAPVD1. Our results indicate that GAPVD1 is an important interacting partner and substrate of CK1δ/ε for endocytosis.

## Introduction

The casein kinase 1 (CK1) family of serine/threonine protein kinases is evolutionarily conserved from yeast to human^1^. Characterized by a conserved catalytic domain, CK1 enzymes vary in the length and sequence of their non-catalytic C-termini^2^. CK1s typically recognize substrates with acidic motifs N-terminal to phosphorylation sites and tend to generate clusters of consecutive phosphoserines on their substrates^3^.

Seven CK1 family members exist in humans (α, α2, δ, ε, γ1-3). CK1α, CK1α2, CK1δ and CK1ε are soluble, while CK1γ1-3 attach to cell membranes via C-terminal prenylation^4–7^. Though the four soluble CK1s are highly related, CK1δ and CK1ε (hereafter referred to as CK1δ/ε) have the greatest degree of sequence identity within their kinase domains, function in similar biological pathways, and share interacting partners and substrates^1,8–14^. In particular, CK1δ/ε have well-characterized functions in circadian rhythm, ribosome biogenesis, and are involved in endocytosis^1,15–18^. However, the full complement of CK1δ/ε-interacting partners, substrates, and functions is still unknown.

In this study, we used CRISPR/Cas9 technology to individually tag CK1δ and CK1ε at their endogenous loci, allowing us to visualize their localization at endogenous protein levels, purify them, and identify associated proteins. Mass spectrometry (MS) analysis identified 181 interacting proteins, the vast majority of which co-purified with both enzymes. GAPVD1, a guanine nucleotide exchange factor (GEF) that is involved in endocytosis^19^, was one of the most prevalent interacting partners. GAPVD1 contains a GAP-like domain and a GEF domain at its N- and C-termini, respectively, that are separated by a predicted intrinsically disordered region (IDR). GAPVD1 is reported to function in insulin-stimulated glucose intake by acting as a GAP towards two small GTPases, TC10^20^ and Ras^19^, and as a GEF towards Rab5A and Rab31^20–22^. Disruption of the *Caenorhabditis elegans* GAPVD1 ortholog, RME-6, reduced the internalization of bovine serum albumin, while also reducing the volume of vesicles containing Rab5^23^. Furthermore, knock-down of GAPVD1 from HeLa cells results in reduced internalization of transferrin (Tfn) and epidermal growth factor receptor (EGFR)^24^, and the loss of the *Drosophila* ortholog of GAPVD1 results in decreased FITC-albumin intake in nephrocytes^25^. Similar defects in nephrotic function were found in humans with homozygous GAPVD1 mutations^25^. An association between GAPVD1 and CK1δ/ε was identified previously, also through affinity purifications and MS analysis^13,14^, but the functional relevance of this interaction has not been previously reported.

Here, we demonstrate that GAPVD1 is not only associated with CK1δ/ε but is also a very good substrate, containing ~38 CK1 phosphosites within its IDR. Eliminating these phosphorylation sites inhibits GAVD1’s endocytic function while a phosphomimetic version of GAPVD1 functions normally. Thus, our results indicate that one way in which CK1δ/ε modulates endocytosis is through phosphoregulation of GAPVD1.

## Results

### Characterization of CK1δ/ε gene-edited HEK293 cells

We used a single round of CRISPR/Cas9-mediated gene editing to individually tag endogenous CK1δ and CK1ε with the multifunctional Venus-MAP (VM) that contains a Flag-streptavidin-His_6_ insert into a loop of the Venus protein^26^ or mNeonGreen (mNG)^27^ in HEK293 cells (Supplementary Fig. 1A,B). CSNK1E encodes a single CK1ε isoform, while CSNK1D encodes two CK1δ isoforms that differ in their C-terminus due to differential splicing^14^. The longer CK1δ form was tagged. In both cases, sequences encoding the tags were placed between the final coding exon and 3’ UTR (Supplementary Fig. 1A). We verified that all alleles in the selected clones had been modified to produce CK1δ-VM, CK1ε-VM, CK1δ-mNG, or CK1ε-mNG by PCR amplifications of 1000 base-pair regions flanking the insert sites of VM or mNG (Supplementary Fig. 1B). Using antibodies that recognize CK1δ or CK1ε, we confirmed that the desired tagging had occurred by immunoblotting whole cell lysates (Supplementary Fig. 1C).

Because deletion of mouse CSNK1D results in embryonic lethality^17,28^, we examined whether tagging CK1δ or CK1ε impaired cell proliferation. We found that there was no change in the rate of cell proliferation of homozygous CK1δ^VM/VM^, CK1ε^VM/VM^, CK1δ^mNG/mNG^, or CK1ε^mNG/mNG^ HEK293 cell lines (Supplementary Fig. 1D).

**Figure 1:**
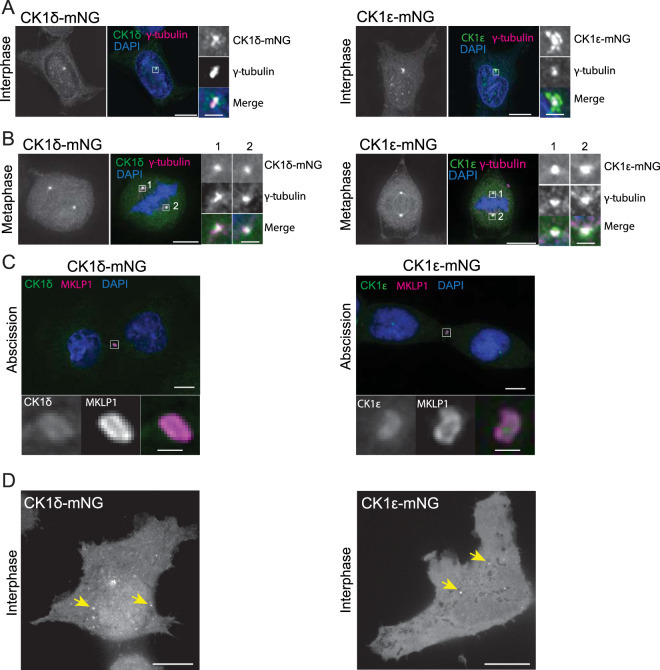
Intracellular localization of endogenous CK1δ-mNG and CK1ε-mNG. (**A–C**) Representative images of fixed HEK293 cells at indicated cell cycle stages producing CK1δ-mNG or CK1ε-mNG stained with (**A**) DAPI and anti-γ-tubulin, (**B**) DAPI and anti-γ-tubulin, or (**C**) DAPI and anti-MKLP1 antibodies. Scale bars, 10 μm. Insets correspond to centrosomes in A and B or the midbody in C. Scale bars, 0.5 μm. (**D**) Representative single z-sections of live-cell images of HEK293 CK1δ-mNG and CK1ε-mNG cells. Yellow arrows indicate examples of vesicle-like structures. Scale bars, 10 μm.

Fixed-cell imaging showed diffuse and punctate localization of both CK1δ-mNG and CK1ε-mNG in the cytoplasm, and diffuse localization in the nucleus of interphase cells (Fig. 1A). Prominent localization to the centrosome was detected throughout the cell cycle (Fig. 1A–D), similar to previous observations based on overexpression of the tagged enzymes in a variety of cell lines^14,29–31^. In addition, we detected these enzymes at the site of abscission marked by MKLP1 staining, a location not previously reported (Fig. 1C). By live cell imaging, many of the cytoplasmic puncta of CK1δ-mNG and CK1ε-mNG (Fig. 1A,D) were mobile (Movie S1). Given the known role of CK1δ/ε in endocytosis^18^, at least a portion of these moving puncta are likely to be endocytic vesicles.

### Identification of CK1δ/ε-interacting partners in HEK293 cells

 We used the cell lines producing CK1δ-VM and CK1ε-VM to identify CK1δ/ε interacting proteins. CK1δ-VM and CK1ε-VM (or VM protein alone as a negative control) were each purified in duplicate from asynchronously growing or mitotic cells, and the purifications were analyzed by liquid chromatography-tandem mass spectrometry (LC-MS/MS). Proteins identified in the VM-only sample (Supplementary Table 1A) were excluded from the list of potential CK1δ/ε interactors. The resulting list of candidate interactors for each CK1 enzyme was filtered further by considering only those proteins identified by a minimum of 10 total spectral counts in each purification. Next, a normalized spectral abundance factor (NSAF) was calculated for each protein^32^ which takes into account the total spectral count and size of each identified protein in relation to the entire data set. Using these criteria, 181 proteins co-purified with either CK1δ or CK1ε (Supplementary Table 1B). Interestingly, 118 proteins, including all of the most abundant proteins, were found in both the CK1δ-VM and in CK1ε-VM purifications, indicating that these enzymes have many interacting partners in common (Supplementary Table 1C). The proteins unique to either enzyme purification were of low abundance and the majority of these were found in CK1δ purifications (51 versus 30) (Supplementary Table 1D,E). Additionally, there was significant overlap between the identified proteins from asynchronous and mitotic cells that co-purified with CK1δ-VM and CK1ε-VM (Supplementary Table 1B), indicating that the primary interacting partners of CK1δ and CK1ε are consistent throughout the cell cycle, or that we failed to capture more labile interactions that distinguish CK1δ/ε functions at different cell cycle stages.

**Figure 2:**
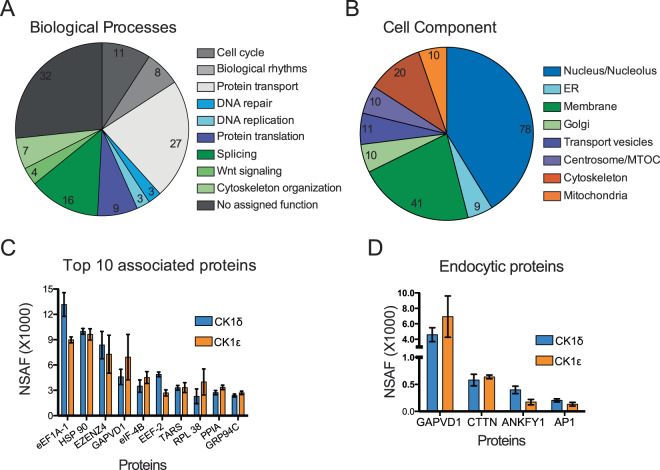
Analysis of CK1δ/ε interacting proteins. (**A**) Pie chart depicting the number of associated proteins with a gene-ontology (GO) annotation^51,52^ that function in general cellular processes. Note that this chart includes 120/181 of the associated proteins. (**B**) Pie chart indicating the number of associated proteins with a GO annotation for localization to a specific cellular compartment. (**C**) The mean NSAF scores for the top ten proteins identified in four affinity purifications each of CK1δ and CK1ε. (**D**) NSAF values of the four identified proteins with a GO annotation for function in endocytosis.

As expected from the pleiotropic functions assigned to CK1δ/ε^8,9,33^, the proteins associated with CK1δ/ε are involved in an array of biological processes including protein transport, circadian rhythm, DNA repair, and cell division (Fig. 2A). Although the most striking localization of endogenously tagged CK1δ/ε enzymes during all stages of the cell cycle is to the centrosome (Fig. 1A,B), centrosomal scaffolding proteins were not among the most abundant interacting proteins, and we did not identify the previously reported centrosomal anchor of CK1δ, AKAP450^34^. A significant subset of co-purifying proteins localize to endosomes and the Golgi apparatus, indicative of the involvement of CK1δ/ε in vesicular trafficking (Fig. 2B)^18,24^. Indeed, one of the most abundant CK1δ/ε interacting proteins was GTPase-containing and VPS9 domain-containing protein 1 (GAPVD1) (Fig. 2C,D).

**Figure 3:**
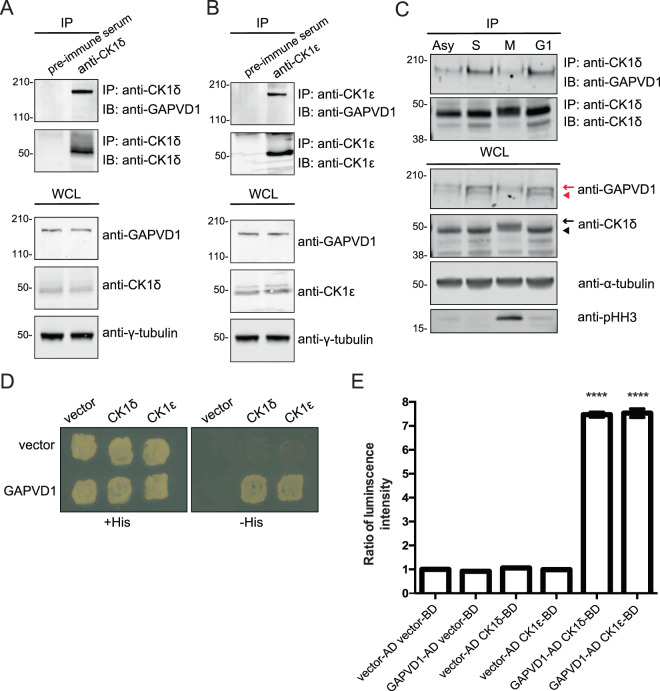
CK1δ/ε interact with GAPVD1 throughout the cell cycle. (**A**,**B**) Immunoblots of the indicated proteins from whole cell lysates (WCL) or immunoprecipitates (IPs) of CK1δ (**A**) or CK1ε (**B**) from HeLa cell lysates. (**C**) Immunoblots of the indicated proteins from WCL (bottom panels) or IPs of CK1δ/ε from asynchronously (Asy) growing HeLa cells or HeLa cells synchronized in S-phase (S), metaphase (M), or G_1_-phase (G1). The black arrow and arrow-head indicate phosphorylated and dephosphorylated CK1δ/ε, respectively. pHH3, phosphohistone H3; a marker of mitotic cells. The red arrow and arrow-head indicate phosphorylated and dephosphorylated GAPVD1, respectively. (**D**) Yeast-two-hybrid showing direct interaction between GAPVD and CK1δ and CK1ε as indicated by growth on -His plates. (**E**) Bar graphs show β-galactosidase activity of the indicated bait and prey plasmids tested for growth in D (represented as ratio to empty bait and prey luminescence intensity). Each assay was performed in triplicate. ****p < 0.001 determined using a one-way ANOVA followed by Tukey’s posthoc test. ns, not significant. Error bars represent SEM. The entire images of immunoblots are presented in Supplementary Fig. 3. In some cases, membranes were cut for hybridization with multiple antibodies and then reassembled.

To validate this interaction in another cell line, we switched to HeLa cells for ease of cell cycle synchronization and assaying endocytosis. We found that GAPVD1 co-immunoprecipitated with CK1δ/ε from HeLa cell lysates when CK1δ/ε were isolated with polyclonal antibodies specific to CK1δ (Fig. 3A) or CK1ε (Fig. 3B). Because our MS results indicated that GAPVD1 is associated with CK1δ/ε in both asynchronous and mitotically arrested cells, we tested whether any changes in their interaction occurred during the cell cycle. Congruent with the MS results, GAPVD1 and CK1δ co-immunoprecipitated from cells arrested at multiple cell cycle stages (G1, S and M) (Fig. 3C). Mitotic cells were validated by the presence of phosphohistone H3 and interestingly, CK1δ appeared to be hyperphosphorylated at this stage (Fig. 3C). In addition, GAPVD1 and CK1δ/ε interact in a yeast-two hybrid assay, suggesting that GAPVD1 and CK1δ/ε may directly interact (Fig. 3D,E).

### GAPVD1 is a CK1δ/ε substrate *in vitro*

**Figure 4:**
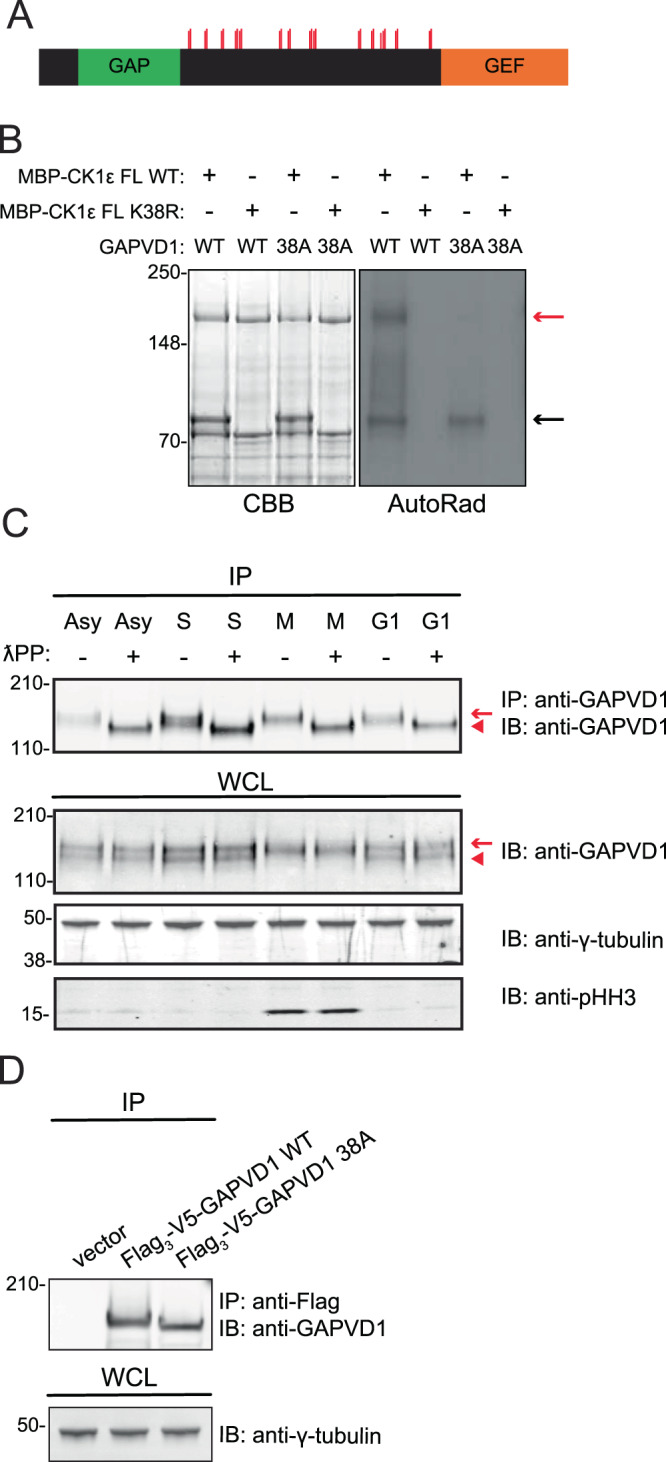
GAPVD1 is a substrate of CK1ε. (**A**) Cartoon representation of GAPVD1 protein domains. Green relates to the GAP domain, orange relates to the GEF domain. Red lines correspond to positions of serine or threonine residues that are mutated to alanine in the GAPVD1 38A mutant. (**B**) *In vitro* kinase assays of recombinant MBP-CK1ε WT and K38R (kinase-dead) detected by Coomassie brilliant blue (CBB) staining of SDS–PAGE gels, with GAPVD1 WT and 38 A as substrates. Phosphorylated GAPVD1 was detected by autoradiography (AutoRad). Black arrow indicates autophosphorylation of MBP-CK1ε. Red arrow indicates GAPVD1. (**C**) Immunoblots of WCLs and IPs of GAPVD1 from asynchronously (Asy) growing HeLa cells or HeLa cells synchronized in S-phase (S), metaphase (M), or G_1_-phase (G1). IPs were treated (+) or not (−) with lambda phosphatase (λPP) and blotted with the indicated antibodies. Red arrow and arrow-head indicate phosphorylated and dephosphorylated GAPVD1, respectively. pHH3, phosphohistone H3; a marker of mitotic cells. (**D**) Immunoblots of IPs from HeLa GAPVD1^−/−^ cells transfected with vector only or vector expressing Flag_3_-V5-GAPVD1 or Flag_3_-V5-GAPVD1-38A. The entire images of immunoblots are presented in Supplementary Fig. 4. In some cases, membranes were cut for hybridization with multiple antibodies and then reassembled.

We next examined if GAPVD1 is a substrate of CK1δ/ε. Our MS experiments and those of other labs^35–38^ revealed that GAPVD1 is highly phosphorylated, predominantly in the IDR, and most of the phosphorylation sites match the consensus motif for CK1δ/ε^39–41^. Our LC-MS/MS analyses of CK1δ/ε-VM purifications identified six sites of GAPVD1 phosphorylation on CK1δ/ε consensus sites (Supplementary Table 1F). However, our experimental method did not include a phosphopeptide enrichment step, limiting our ability to identify the full cohort of phosphorylated residues. For this reason, we mutated all 38 serine and threonine residues to alanine in the IDR of the protein that fit the consensus motif for CK1δ/ε and were identified using MS, either by us or others^36,42,43^ (Fig. 4A). We found that wildtype recombinant MBP-CK1ε but not a kinase-dead MBP-CK1ε phosphorylated the wildtype GAPVD1, but not GAPVD1-38A (Fig. 4B). Consistent with the cumulative MS data detecting 38 phosphorylated residues clustered in the IDR of GAPVD1, and similar to the ‘strings’ of serines and threonines phosphorylated in other substrates^3^, we found that mutating only the subset of the 38 sites identified in our purifications (*S566, S569, S740, S742, S746, S747, S902, S903)* did not eliminate CK1ε-mediated phosphorylation of GAPVD1 *in vitro* (data not shown).

As expected for a highly phosphorylated protein, GAPVD1 immunoprecipitates treated with lambda phosphatase migrated faster on SDS-PAGE than untreated immunoprecipitates, irrespective of cell cycle stage (Fig. 4C). Mitotic cells were validated by blotting with antibodies to phosphohistone H3 (Fig. 4C). When transiently transfected into HeLa cells, immunoprecipitated, and analyzed by immunoblotting, Flag_3_-V5-GAPVD1-38A migrated faster than Flag_3_-V5-GAPVD1 WT, consistent with the loss of phosphorylation *in vivo* (Fig. 4D).

### GAPVD1 phosphorylation promotes endocytosis

**Figure 5:**
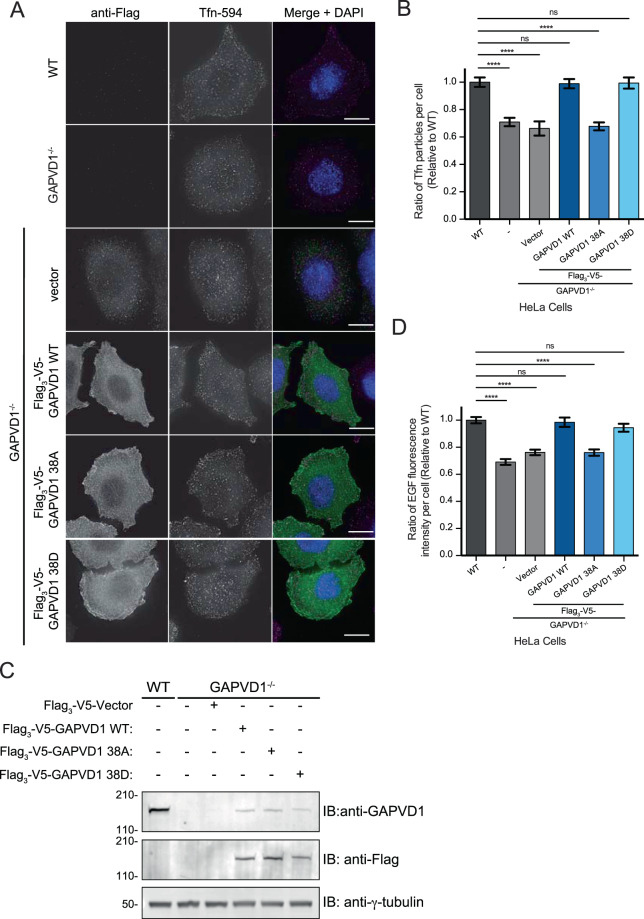
GAPVD1 phosphorylation promotes endocytosis. (**A**) Representative images of HeLa WT and GAPVD1^−/−^ cells incubated with Tfn-594 in an endocytosis assay. Scale bar 10 μm. (**B**) Quantification of Alexa 594-coupled Tfn uptake in HeLa WT and GAPVD1^−/−^ cells transfected with Flag_3_-V5 plasmids. The mean and SEM from 3 independent experiments (n ≥ 40 cells per experiment) are presented as a ratio to control. (**C**) Immunoblots of whole cell lysates from the experiments in (**A**,**B**). (**D**) Quantification of Alexa 488-coupled EGF uptake in HeLa WT and GAPVD1^−/−^ cells transfected with Flag_3_-V5 plasmids. The mean and SEM from 3 independent experiments (n ≥ 40 cells per experiment) are presented as a ratio to control. In (**B**,**D**), ****p < 0.001, *p* values determined using a one-way ANOVA followed by Tukey’s posthoc test. ns, not significant. The entire images of immunoblots are presented in Supplementary Fig. 5. In some cases, membranes were cut for hybridization with multiple antibodies and then reassembled.

GAPVD1 has reported roles in fluid-phase and receptor-mediated endocytosis and CK1δ/ε are also required for efficient Tfn and EGFR internalization^18,24^, an endocytic role conserved with the CK1δ/ε ortholog Hrr25 in *Saccharomyces cerevisiae*^18^. Thus, we established cell lines in which we could test the effect of CK1 on GAPVD1 function. The GAPVD1 gene was disrupted in HeLa cells using CRISPR/Cas9-mediated gene editing (Supplementary Fig. 2A). The consequent loss of all copies of GAPVD1 from three independently isolated cell lines was validated by immunoblotting (Supplementary Fig. 2B). To confirm that loss of GAPVD1 resulted in a defect in endocytosis, the internalization of Tfn conjugated to Alexa-594 (Tfn-594) was assayed in GAPVD1^−/−^ cells as previously described^44^. In all three GAPVD1^−/−^ cell lines, we detected a reduction in the internalization of Tfn-594, consistent with a general endocytic defect (Supplementary Fig. 2C). Furthermore, the observed endocytic defect could be rescued by re-introducing GAPVD1 (Fig. 5B and Supplementary Fig. 2D,E).

We next asked if phosphorylation at CK1 sites modulates GAPVD1’s role in endocytosis by transfecting into the GAPVD1^−/−^ cell lines WT GAPVD1, the 38 A mutant of GAPVD1, or a mutant of GAPVD1 in which the 38 phosphosites were replaced with aspartic acid residues (38D), and then measuring either Tfn uptake as described above or Epidermal Growth Factor (EGF) uptake as described previously^24^ (Fig. 5). Although expressed to an equivalent level as WT, the 38 A mutant of GAPVD1 did not rescue the endocytosis defects of GAPVD1^−/−^ cells (Fig. 5B-D). In contrast, GAPVD1-38D did rescue the endocytic defects of these cells (Fig. 5B-D). These results indicate that GAPVD1 phosphorylation by CK1δ/ε promotes endocytosis.

## Discussion

The full complements of interacting partners and substrates of CK1δ/ε have not been well defined. Although this study is not the first to investigate the localization or interactome of CK1δ/ε^13,14^, we are the first to use CRISPR/Cas9 gene editing to tag and purify these endogenous enzymes, which alleviates any issue that could arise from overexpression studies. We confirmed all previous noted localization patterns for CK1δ/ε (centrosome, nucleus, cytoplasm)^9^ and discovered that CK1δ/ε also localize to the midbody. In addition to identifying known interacting partners involved in biological rhythms, such as Period protein 1 and 2^13,15,45,46^, we identified additional interacting proteins involved in other biological processes that CK1δ/ε are involved in, such as endocytosis. Twenty-seven of the identified proteins are involved in protein transport, with four having roles specific to endocytosis. Although CK1δ/ε and their *Saccharomyces cerevisiae* homolog, Hrr25, have been shown to localize to sites of clathrin-mediated endocytosis (CME)^18^ and loss of CK1δ or CK1ε significantly reduces passive and ligand-stimulated CME^18,24^, CK1 substrates involved in CME have only been identified in yeast^18^. Our analysis has shown that CK1δ/ε phosphorylation of GAPVD1 is key to its role in CME. In addition to GAPVD1, our list of interacting proteins contains good candidates for additional endocytic CK1δ/ε substrates in human cells. Indeed, our study provides a plethora of information for further study of the involvement of CK1δ/ε in multiple biological processes.

The most striking localization of endogenously tagged CK1δ/ε enzymes during all stages of the cell cycle is to the centrosome. Due to the many contributions of the centrosome to mitosis^47,48^, we sought to identify the interacting partners of CK1δ/ε not only in asynchronously growing cells but also in mitotic cells but centrosomal proteins were surprisingly not abundant co-purifying proteins. One potential explanation for this result is that CK1δ/ε centrosomal localization may be very dynamic, and transient interactions are difficult to detect by co-purification approaches. Another unexpected result from our MS data is that there were few co-purifying proteins specific to either asynchronous or mitotic samples, which might indicate either that interaction partners do not change appreciably during the cell cycle or that we failed to capture more labile interactions that distinguish CK1δ/ε functions at different cell cycle stages.

Similar to other substrates of CK1δ/ε^3^, there are many sites of phosphorylation on GAPVD1. The 38 CK1 consensus phosphorylation sites are clustered in the IDR of GAPVD1 similar to the ‘strings’ of serines and threonines phosphorylated in other substrates^3^. Many questions remain about GAPVD1 phosphorylation including whether all of these sites need to be phosphorylated in order to support GAPVD1 function in endocytosis. It also remains to be determined whether there is a specific order in which phosphorylation of GAPVD1 occurs and whether some sites are more prevalent or important than others. Though the GAPVD1 GEF domain has been shown to bind Rab5 via yeast two-hybrid^23^ and act as a GEF toward Rab5A *in vitro*^19^, GEF or GAP activity of purified full-length GAPVD1 has never been reported nor has it been shown to interact directly with another protein. Thus, it will also be interesting to determine how phosphorylation impacts GAPVD1 function, whether by stimulating its GEF domain, modulating the function of its GAP-like domain, and/or influencing other reported interactions^21,22,25^.

While no other kinase has been shown to phosphorylate and regulate GAPVD1 function, other GAPVD1 phosphosites have been identified in phosphoproteomic screens that fit Cdk1, p38 and PKA consensus motifs^36,42,43^. CK1δ/ε phosphorylation of GAPVD1 may thus be dependent upon and is likely coordinated with phosphorylation by other kinases as in the case for YAP in the Hippo pathway^12^.

## Material and methods

### Molecular biology methods

GAPVD1 cDNA encoding isoform 6 was purchased from Dharmacon (Cat#: MHS6278-211690496). Because this isoform lacks amino acids 1057-1074 relative to the previously studied isoform 1^22^ and also has an insertion at amino acid 810 (DFLYILQPKQHFQHIEAEADMRIQLSSS), a geneBlock fragment from Integrated DNA Technology was used to construct the longer isoform. GAPVD1 plasmids were constructed using Gateway cloning, in the case of Flag_3_-V5-ccdB (Addgene #87064). Gibson cloning was used for construction of peGFP-C2-GAPVD1.

The ORF of CK1δ was amplified by PCR from a plasmid (CK1δ pGEX-6p-2) kindly provided by Fanni Gergely (University of Cambridge). The CK1ε ORF was amplified by PCR from GST-CK1ε. Each PCR product was cloned into pMAL-C2, and the correct sequence was validated by DNA sequencing.

For CRISPR/Cas9 gene editing, single guide RNAs (gRNA) were designed using the algorithm designed by the Zhang lab^49^ at crispr.mit.edu. The gRNAs were cloned into pSpCas9(BB)-2A-Puro (Addgene, PX459). Repair plasmids for knock-in gene editing were constructed using pUC19 as a backbone, with restriction sites BamHI and EcoRI. The regions flanking the tags were amplified using purified genomic DNA from HEK293 cells using the Qiagen DNeasy blood and tissue kit (Qiagen, Venlo, Netherlands). Repair plasmids were constructed using Gibson cloning, with at least 25 base pairs of overlapping sequence between each of the adjacent fragments. For insertion of the blasticidin resistance gene, a geneBlock fragment consisting of a ribosomal skip sequence followed by the blasticidin resistance gene was purchased from IDT.

### Cell culture and gene editing

HeLa and HEK293 cells were cultured in Dulbeco’s modified eagle medium supplemented with 10% fetal bovine serum and 1% penicillin/streptomycin. For CRISPR-mediated gene-editing, cells were transfected with gene editing reagents as described previously^49^. Briefly, for knock-in cell line generation, 6 × 10^5^ cells were transfected with 300 ng of CRISPR/sgRNA plasmid and 1.5 μg of repair plasmid using Lipofectamine 3000. 24 hours after transfection, 5 μg/mL of puromycin was added for an additional 48 hours. For creating GAPVD1 knock-out cell lines, cells were further treated with 5 μg/mL blasticidin for 48 hours. Following selections, cells were transferred to a 10-cm dish without selection for 24 hours. Cells were then single-cell sorted through the Vanderbilt Flow Cytometry core facility into 10 96-well plates for each knock-in cell line and a single 96-well plate was filled for the knock-out cell lines. Clones were validated through analysis of whole-cell lysates via immunoblotting and PCR amplification of genomic DNA and DNA sequencing. Genomic DNA was extracted using the Qiagen DNeasy blood and tissue kit, following the manufacturer’s instructions.

For gene rescue experiments, cells were transfected with 2 µg of plasmid DNA using the Nucleofection R kit according to manufacturer’s instructions (Lonza) using an AMAXA instrument with their program I-013.

### Cell cycle synchronization

HeLa and HEK293 cells were synchronized using a sequential thymidine (Sigma-Aldrich) and aphidicolin (Tocris Bioscience) block-and-release protocol with drugs used at 2.5 mM and 5 μg/mL, respectively. To capture metaphase cells, cells were released from the second block for eight hours, then treated with 25 μM proTAME (R&D Systems) and 100 μM Apcin (R&D Systems) for four hours. Mitotic enrichment was verified by analyzing cells using phase-contrast microscopy and measuring the percent of rounded cells. The presence of the mitotic antigen, phosphoH3 (Millipore Sigma) was also used for validating a mitotic cell population. For G1 synchronization, cells were released from the metaphase arrest for 3-4 hours.

### Protein methods

Cell lysates were prepared as previously described^29^. Briefly, cell pellets were lysed in 1% NP-40 buffer containing 6 mM Na_2_HPO_4_, 4 mM NaH_2_PO_4_, 1% Nonidet P40, 150 mM NaCl, 2 mM EDTA, 50 mM NaF, 0.1 mM Na_3_VO_4_ and supplemented with 1 mM PMSF, benzamidine, sodium orthovanadate, β-glycerophosphate, sodium fluoride, and sodium pyrophosphate. For immunoblotting, IP samples or ~40 μg of WCL was resolved by SDS-PAGE on 4-12% or 3-8% gels and transferred by electroblotting to PVDF membrane (Immobilon P; Millipore, Bedford, MA). Proteins were immunoprecipitated with anti-GAPVD1 (Bethyl Laboratoy; 2 μg), anti-FLAG (M2; Sigma-Aldrich) or a serum raised and purified against GST-CK1δ (amino acids 1-415, VU477, Cocalico Biologicals, 2 μg) or serum raised against GST-CK1ε (amino acids 1-416, VU473, Cocalico Biologicals, 2 μg). Immunoblots were visualized using the default settings of Odyssey software (LI-COR Biosciences).

Antisera were raised against recombinant GST-CK1δ or GST-CK1ε (Cocalico), and their specificity was verified by immunoblotting. The anti-CK1δ/ε serum was further purified by ammonium sulfate precipitation. The serum was cleared by centrifugation and then precipitated with 0.5 volumes of saturated ammonium sulfate added dropwise and incubated overnight at 4 °C. The precipitate was cleared from the serum by centrifugation and then the serum was precipitated with an additional 0.5 volumes of saturated ammonium sulfate added dropwise and incubated overnight at 4 °C. The precipitate was pelleted by centrifugation, resuspended in 0.4 volumes phosphate-buffered saline (PBS), and dialyzed three times in PBS.

For multifunctional affinity purifications, CK1δ/ε were purified as described previously^26^. Briefly, twenty 10 cm plates of HEK293 cells at ~80% confluence were released from plates with trypsin and washed three times with ice cold PBS. Cell pellets were then lysed in 3 mL of lysis buffer containing 50 mM Tris-HCl pH7.5, 150 mM NaCl, 0.5% Triton X-100, 5% glycerol, 1 mM EDTA, and protease and phosphatase inhibitors (1 mM PMSF, benzamindine, sodium othrovanadate, β-glycerophosphate, sodium fluoride, and sodium pyrophosphate). Lysates containing ~35 mg of protein were incubated at 4 °C on a nutator for 20 minutes, followed by centrifugation at 16,000xg for 15 minutes at 4 °C. Lysate was incubated with 150 μL of anti-Flag magnetic beads (Sigma-Aldrich) at 4 °C for 2 hours. The magnetic beads were washed three times with wash buffer (50 mM Tris-HCl, pH7.5, 250 mM NaCl, 0.05% NP-40). Bait protein was then eluted using 300 μg/mL of 3X-Flag peptide (Sigma-Aldrich) in 2 mL of lysis buffer. The eluted sample was then incubated in 300 μL of streptavidin-coated beads for 2 hours at 4 °C. The beads were washed two times with wash buffer, transferred to a 10 mL column and washed once with elution buffer (25 mM Tris-HCl, pH 7.5, 125 mM NaCl). Proteins were eluted two times with 1 mL of 2.5 mM biotin in elution buffer. The eluates were TCA precipitated by adjusting them to 25% TCA with 100% TCA, placing them on ice for 30 min with periodic vortexing, and spinning at maximum speed for 30 min at 4 °C. The TCA pellets were washed once with cold (−20 °C) acetone containing 0.05 N HCl and spun for 7 min at maximum speed at 4 °C. They are then washed once with cold (−20 °C) acetone and spun for 7 min at maximum speed at 4 °C. The supernatant was removed and the pellets dried in a speed-vac.

### Endocytosis assay

Endocytosis assays were performed as described previously^44^. Specifically, cells were washed with PBS and then serum starved with DMEM supplemented with 0.5% FBS, 1% Pen/Strep (low-serum media) for two hours at 36 °C, 5% CO_2_. Cells were incubated with 30 μg/uL transferrin-594 (Tfn-594, Thermo Fisher Sci, cat# T13343) or 100 ng/mL epidermal growth factor-488 (EGF-488 Thermo Fisher Sci, cat# E13345) in low serum media for 30 minutes on ice and covered with tin foil. Cells were washed twice with PBS, once with low-serum media, and incubated in low-serum media with 300 μg/mL Tfn or 200 ng/mL EGF (Sigma-Aldrich, cat# T8158) for 10 minutes at 36 °C, 5% CO_2_. Cells were then acid washed four times for one minute per wash. Cells were washed with PBS twice and fixed with 4% PFA.

### Fixation and antibody staining

For methanol fixation, cells were washed once with cold PBS and once with 100% cold methanol, followed by fixation with 100% cold methanol for 15 minutes at −20 °C. Cells were then washed 3X with 1x phosphate buffered saline +0.1% Tween-20 (0.1% PBST) at room temperature, followed by blocking with 2% normal goat serum in 0.1% Triton X100 in 1x PBS for 10 minutes. For paraformaldehyde (PFA) fixation, cells were washed twice with PBS, once with 4% PFA, then fixed with 4% PFA for 15 minutes at room temperature. Cells were incubated with antibodies against γ-tubulin (Sigma-Aldrich, GTU88; 1:500), Flag M2 (Sigma-Aldrich, 1:1500), or MKLP1 (Bethyl laboratory, 1:1000). Cells were then incubated with DAPI and secondary antibodies (Alexa Fluor Goat-anti Mouse or Rabbit IgG (H + L), ThermoFisher; 1:500) for 45 minutes at 22 °C. Coverslips were mounted on slides using Prolong Gold antifade mounting media.

### Microscopy methods (equipment settings included)

Fixed-cell images of HeLa and HEK293 cells were acquired using a personal DeltaVision microscope system (Applied Precision) that includes an Olympus IX71 microscope, 60 × NA 1.42 PlanApo and 100 × NA 1.40 UPlanSApo objectives, a Photometrics CoolSnap HQ2 camera, and softWoRx imaging software. All Images in figures were acquired at a pixel size of 1024 × 1024, with z-sections spaced at 0.2 µm, 1 × 1 binning. TRITC, FITC and DAPI filters were used. All images were deconvolved with 10 iterations. Quantitative analysis of microscopy data was performed using Fiji (a version of ImageJ software) available at: https://fiji.sc/^50^. The mean number of Tfn-594 particles per cell was determined by first setting an intensity threshold for each of the control cells. This threshold was then used in the ‘3D object counter’ plug-in for all of the images. The depth for fixed- and live-cell images fanged from 4-8 µm

For live-cell imaging a Nikon spinning disc microscope was used at the Nikon Center of Excellence in Vanderbilt. The system consists of a Yokogawa CSU-X1 spinning disk head, Andor DU-897 EMCCD camera, a high-speed piezo [z] stage, and live cell incubator set to 37 °C with 5% CO_2_. A Plan Apo Lambda (oil) 60 × 1.40 NA WD 0.13 mm was used for image acquisition. Images were acquired at a pixel size of 512 × 512. A 488 nm, 65 mW diode laser line was used to visualize CK1δ^mNG/mNG^ CK1ε^mNG/mNG^ HEK293 cells. 500 msec exposure every 30 seconds for 5 minutes. Cells were imaged in standard tissue culture media for HEK293 cells (DMEM supplemented with 10% FBS and 1% P/S).

### *In vitro* kinase assays

Bacterial expression of MBP-CK1 fusion proteins were performed in terrific broth media at an OD_595_ of ~1.2 and 0.4 μM isopropyl β-D-1-thiogalactopyranoside. MBP-CK1 fusion proteins were purified on amylose beads (New England Biolabs) in column buffer (20 mM Tris [pH 7.0], 150 mM NaCl, 2 mM EDTA, 1 mM DTT and 0.1% NP40) and eluted with maltose (10 mM). Kinase reactions were performed with 1000 ng kinase, 1000 ng GAPVD1, 10 μM ATP plus 1 µCi γ-[^32^P]-ATP in kinase buffer (50 mM Tris [pH 7.5], 10 mM MgCl_2_, and 5 mM DTT) in 20 µl at 30 °C for 45 minutes. Reactions were quenched by adding SDS-PAGE sample buffer and proteins were separated by SDS-PAGE. Phosphorylated proteins were visualized by autoradiography and relative protein quantities were assessed by Coomassie blue staining relative to known standards utilizing Odyssey software (LI-COR Biosciences). Phosphorylated proteins were visualized and quantitated using an FLA7000IP Typhoon Storage Phosphorimager (GE Healthcare Life Sciences).

### Two-hybrid analyses

Two-hybrid experiments were performed as described previously (Vo et al., 2016). GAPVD1, CK1δ and CK1ε, cloned into pDEST DB and pDEST AD vectors, respectively, were generously provided by Haiyuan Yu (Cornell University). These were co-transformed into *S. cerevisiae* strain PJ69-4A. Leu+ and Trp+ transformants were selected and then scored for positive interactions by streaking onto synthetic dextrose plates lacking tryptophan, leucine, and histidine. β-Galactosidase reporter enzyme activity in the two-hybrid strains was measured using the Galacto-Star chemiluminescent reporter assay system according to the manufacturer’s instructions (Applied Biosystems, Foster City, CA), except that cells were lysed by glass bead disruption. Each experiment was performed in triplicate. Reporter assays were recorded on a Multi-Detection Microplate Reader (Bio-TEK Instruments).

### Mass spectrometry analysis

TCA-precipitated proteins MAP purifications were subjected to mass spectrometric analysis on an LTQ velos by 3-phase multidimensional protein identification technology as previously described (McDonald et al., 2002; Chen et. al. 2013) with modifications. Proteins were re-suspended in 8 M urea buffer (8 M urea in 100 mM Tris, pH 8.5), reduced with Tris (2-carboxyethyl) phosphine, alkylated with 2-chloro acetamide, and digested with trypsin or chymotrypsin. The resulting peptides were desalted by C-18 spin column (Pierce). Raw mass spectrometry data were filtered with Scansifter and searched by SEQUEST algorithm. Scaffold (version 4.4.8 or version 4.2.1) and Scaffold PTM (version 3.0.1) (both were from Proteome Software, Portland. OR) were used for data assembly and filtering. The following filtering criteria were used: minimum of 90.0% peptide identification probability, minimum of 99% protein identification probability, and minimum of two unique peptides.

## Supplementary information

Supplementary information.

Supplementary information2.

Supplementary Information 3.
