# Supplementary material for: CRISPR-mediated gene targeting of CK1δ/ε leads to enhanced understanding of their role in endocytosis via phosphoregulation of GAPVD1
Source: Sci Rep. 2020 Apr 22;10:6797. doi: 10.1038/s41598-020-63669-2 (PMC7176688; doi:10.1038/s41598-020-63669-2)
Supplement: Supplementary file 3 — Supplementary Information 3. [file 41598_2020_63669_MOESM3_ESM.pdf]

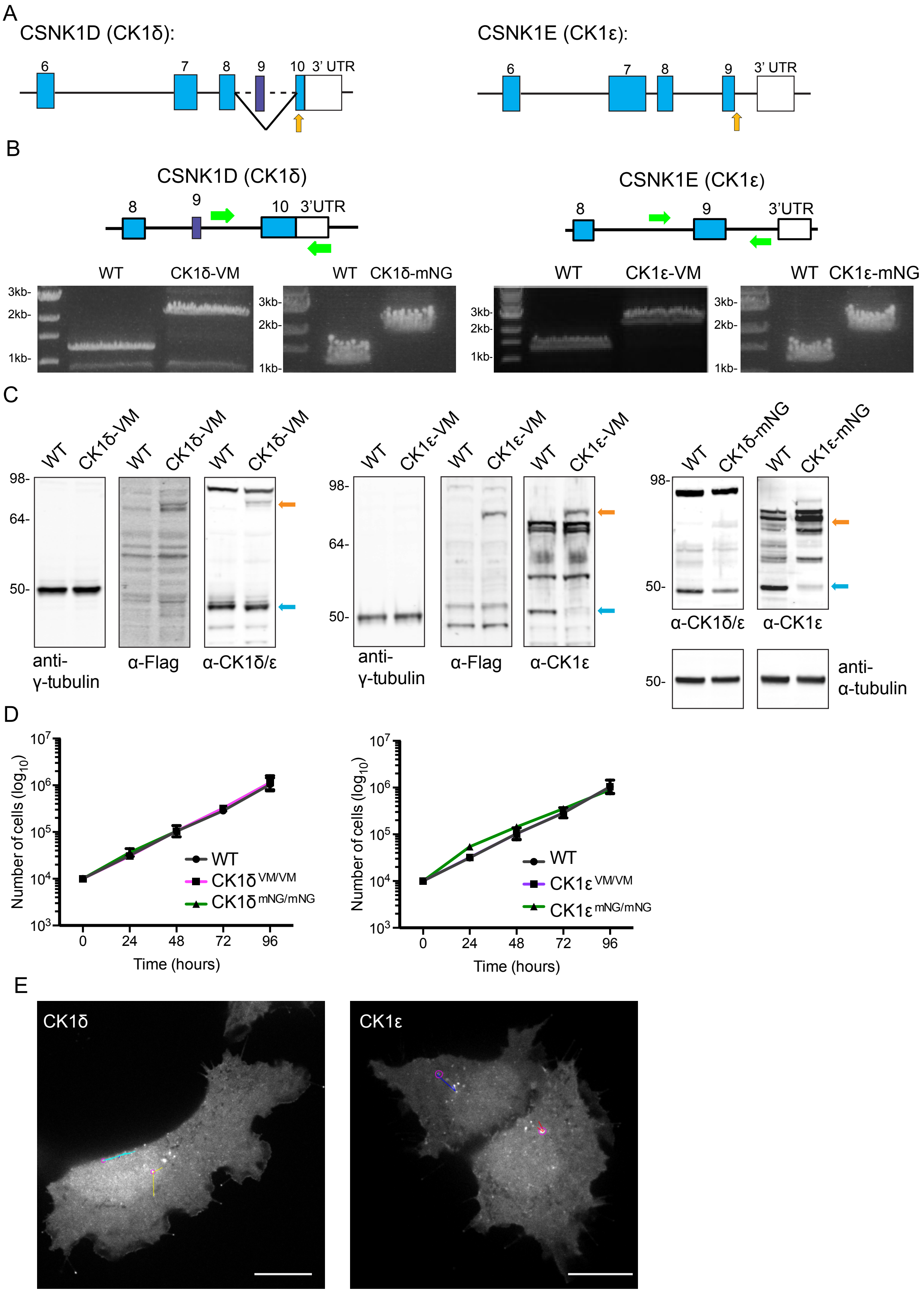

Guillen et al Figure S1

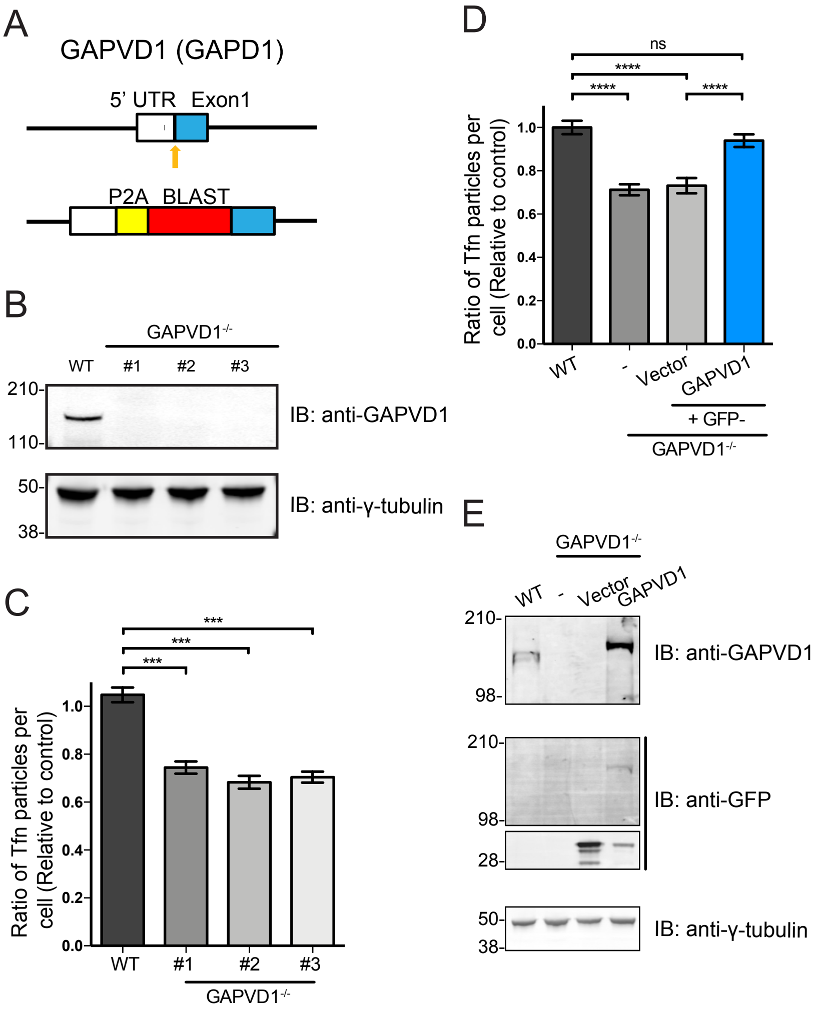

Guillen et al Figure S2

Supplementary Figure legends

Figure S1: CRISPR gene-editing of CK1δ and CK1ε. (A) Cartoon representation of the 3'ends of the CK1δ (CSNK1D) and CK1ε (CSNK1E) genes. The orange arrows point to the positions where gRNAs were designed to target Cas9 for gene editing. Numbers correspond to exons. (B) PCR verification of cell lines with endogenously tagged CK1δ/ε variants. Green arrows indicate positions of primers used to verify insertion of tags by PCR analysis. (C) Immunoblot analyses of whole cell lysates (WCLs) from wildtype (WT) and CK1δ/ε-VM cells with anti-γ-tubulin or anti-α-tubulin (loading control), anti-Flag or anti-CK1δ/ε antibodies. Orange arrows indicate fluorescent fusion proteins. Blue arrows indicate untagged proteins. (D) Proliferation assays of WT HEK293 and CK1δVM/VM, CK1δmNG/mNG, CK1εVM/VM and CK1εmNG/mNG cell lines.

Figure S2: GAPVD1 promotes endocytosis. (A) Cartoon representation of the 5'end of the GAPVD1 (GAPD1) gene and the repair plasmid that disrupts GAPVD1 expression. The orange arrow points to the position where a gRNA was designed to target Cas9 for gene editing. Numbers correspond to exons. BLAST: Blasticidin; P2A: ribosomal skip sequence. (B) Immunoblots of WCLs from WT and GAPVD1$^{-/-}$ cells (#1-3) with anti-GAPVD1 and anti-γ-tubulin (loading control) antibodies. (C) Quantification of Alexa 594-coupled Tf uptake in HeLa WT and GAPVD1$^{-/-}$ cells. (D) Quantification of Alexa 594-coupled Tf uptake in HeLa WT and GAPVD1$^{-/-}$ cells transfected with GFP-C2 vector or GFP-GAPVD1. For C and D, the mean and standard error of the mean (SEM) from 4 independent experiments ($n \geq 40$ cells per experiment) are presented as the ratio to control. ***, $p < 0.005$,****, $p < 0.001$, p values determined using a one-way ANOVA followed by Tukey's posthoc test. ns, not significant. (E) Representative immunoblot of WCLs from cells in D.
